# Using the Health Belief Model to Predict Pre-Travel Health Decisions among U.S.-Based Travelers

**DOI:** 10.4269/ajtmh.22-0633

**Published:** 2023-09-05

**Authors:** Erica Rapheal, Ranjini Prithviraj, Stephanie Campbell, Steven T. Stoddard, Valerie A. Paz-Soldan

**Affiliations:** ^1^ Independent Consultant, Saint Paul, Minnesota;; ^2^ Emergent BioSolutions Inc., Gaithersburg, Maryland;; ^3^ Graduate School of Public Health, San Diego State University, San Diego, California;; ^4^ School of Public Health and Tropical Medicine, Tulane University, New Orleans, Louisiana

## Abstract

International travelers are at increased risk of infectious disease, but almost half of Americans traveling to lower- and middle-income countries seek no health information before traveling. The Health Belief Model (HBM) can help evaluate decisions by categorizing behaviors into five categories: susceptibility, severity, benefits, barriers, and self-efficacy. This study sought to use the HBM to elucidate what may influence an individual to make certain pre-travel health decisions. We surveyed 604 participants who had recently traveled to an at-risk country. Participants were subset into nested groups: full population, sought any health information, and visited a clinic or health care provider (HCP). Survey questions were categorized according to the HBM, assembled into a priori models, and analyzed in each group using logistic regression with three main outcome variables: “Sought any pre-travel health information,” “Visited clinic or HCP,” and “Received vaccine.” Of the 604 participants, 333 (55%) sought any health information, 245 (41% of total) reported visiting an HCP, and 166 (27% of total) reported receiving a vaccine before traveling. Models containing variables from the susceptibility and benefits categories were most successful in predicting all three outcomes; susceptibility was a more relevant consideration in information seeking and seeing a provider than vaccination, whereas benefits was relevant for all outcomes. Our results emphasize the importance of an individual’s perceived susceptibility to disease and perceived benefit of interventions in predicting pre-travel health behaviors. Understanding this interaction can help shape how HCPs and public health entities can encourage health care seeking and vaccine uptake in travelers.

## INTRODUCTION

International travelers are at increased risk of infectious diseases, including vector-borne diseases such as malaria and Zika and food and waterborne diseases such as cholera and enteric fever, particularly when visiting tropical lower- and middle-income countries (LMICs). These travelers also risk bringing home vaccine-preventable diseases (VPDs) that are otherwise rare in the U.S. population, such as typhoid fever, hepatitis A, and measles.^1^ Although information on the overall burden of VPDs in travelers is limited, a large study of ill returning travelers found that around 15 per 1,000 had contracted a VPD while abroad; with almost 100 million international departures from the United States in 2019, this represents a significant risk.^1^^,^^2^ Enteric fever, acute viral hepatitis, and influenza were the most common diseases in that study.^1^

Despite the risk, almost half of Americans traveling to LMICs, where infectious disease risk is generally higher, do not seek any health information before traveling.^3^ Of those who see a health care provider (HCP), 25% refuse at least one recommended vaccine.^4^ Meanwhile, among travelers who present to a clinic with a VPD upon return to the United States, nearly 30% saw an HCP prior to traveling, suggesting that they may have refused a recommended vaccine or were not recommended a vaccine that would have prevented their illness.^5^ Understanding the travel health decision-making pathway from seeking information, to seeing a provider, to receiving a vaccine is critical for developing interventions that encourage travelers to seek care before traveling and increasing travel vaccine uptake.

Barriers to pre-travel care range from lack of information to cost to lack of concern about a given disease.^3^^,^^4^ Vaccine hesitancy in particular poses a significant risk; between 65% and 96% of individuals who refused a vaccine in a 2017 study cited a reason other than cost.^4^ With vaccination and vaccine hesitancy at the forefront of social and political discourse throughout the COVID-19 pandemic, vaccine hesitancy has become an even more significant concern in travel medicine.^6^

**Figure 1.**
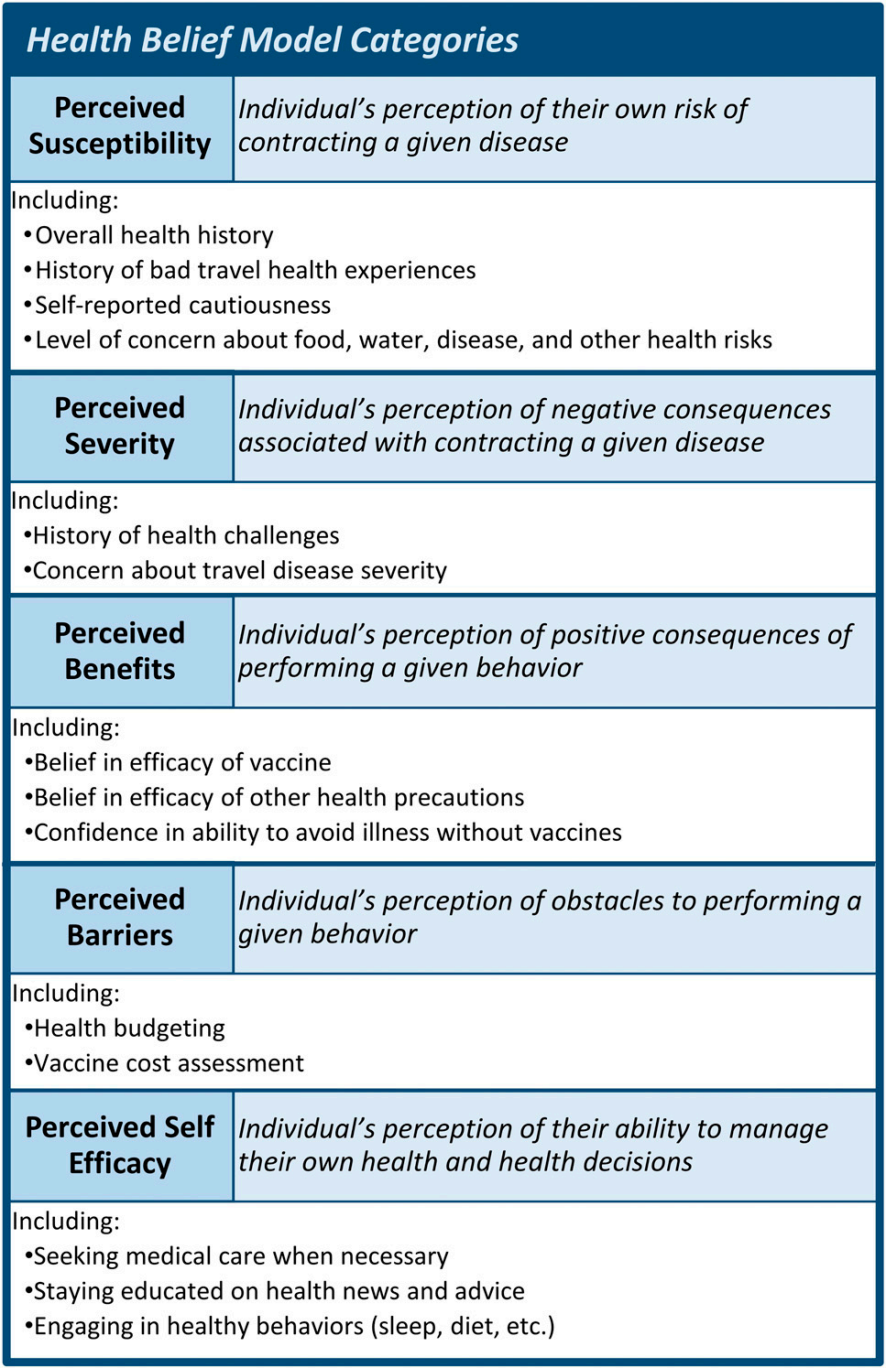
Health Belief Model categories, definitions, and examples of types of variables included in this study.

The Health Belief Model (HBM) provides one way to evaluate the behaviors that influence pre-travel health seeking. Created in the 1950 s to help explain widespread failure to seek preventive health care, the HBM describes five categories that may facilitate or hinder health-related behaviors: perceived susceptibility to disease, perceived severity of disease, perceived benefits of intervention, perceived barriers to intervention, and self-efficacy to seek and receive the intervention (Figure 1).^7^^,^^8^ The HBM has been widely used as a framework to study vaccine hesitancy, but studies specific to travel medicine are limited.^9^^–^^15^ One recent study using the HBM to describe COVID-19 vaccine hesitancy found the susceptibility and benefits categories were most relevant to vaccine uptake, whereas a 2012 study of H1N1 vaccination found barriers to be most relevant.^9^^,^^13^ The HBM is still being studied in the context of travel medicine, and more research is necessary to assess which categories are the best targets for intervention.^9^^,^^11^^,^^12^^,^^14^^,^^15^

This study sought to use the framework of the HBM to help explain why travelers decide to seek health information, see an HCP, and get vaccinated before traveling to LMIC settings.

## MATERIALS AND METHODS

### Traveler survey.

The survey was developed to investigate pre-travel health-seeking behavior among U.S.-based international travelers generally. Participants were recruited from an anonymous panel of millions of voluntary survey participants maintained by Survey Sampling International (now Dynata; www.dynata.com). Within this overall population, we analyzed data collected from adults aged 18–65 years who had traveled to specific international destinations in the prior 3 years. The list of countries included was determined based on travel vaccine requirements and risk for cholera in 2018, as indicated by the CDC and expert opinion. All of the included destinations should have had travel vaccine recommendations, allowing for our assessment of vaccine uptake by international travelers.

Inclusion/exclusion criteria

- Inclusion:
  ○ Residing in the United States
  ○ Aged 18–65 years
  ○ At least one international trip outside of North America (United States, Canada, Puerto Rico, Mexico) in the last 3 years
  ○ Travel to specific countries in Asia, Africa, Middle East, and the Caribbean (Supplemental Table 1).
- Exclusion:
  ○ Work in any of the following areas (participant or household/family member): advertising/public relations, marketing or market research, pharmaceuticals, medicine or medical school, pharmaceutical distributor, pharmacy, media

Survey participation was voluntary and covered five categories: general attitudes toward life and wellness (e.g., “Risk-taking is exciting to me,” “I trust my doctor to tell me what is right for me,”); travel attitudes and behavior (e.g., “How far in advance do you typically book your international personal/leisure trips?”); travel journey, with questions regarding the individual’s most recent trip (e.g., “How many nights did you spend away for your trip?” “What was your planned budget for this trip?”); travel vaccine attitudes and behavior, asking about the individual’s travel health knowledge and vaccine behavior (e.g., “How familiar are you with the following diseases?” “Have you ever received a vaccine for the following diseases?”); and demographics. This survey was developed for market research, and the list of countries included was determined based on travel vaccine requirements without considering public health research implications.

### Statistical analyses.

**Figure 2.**
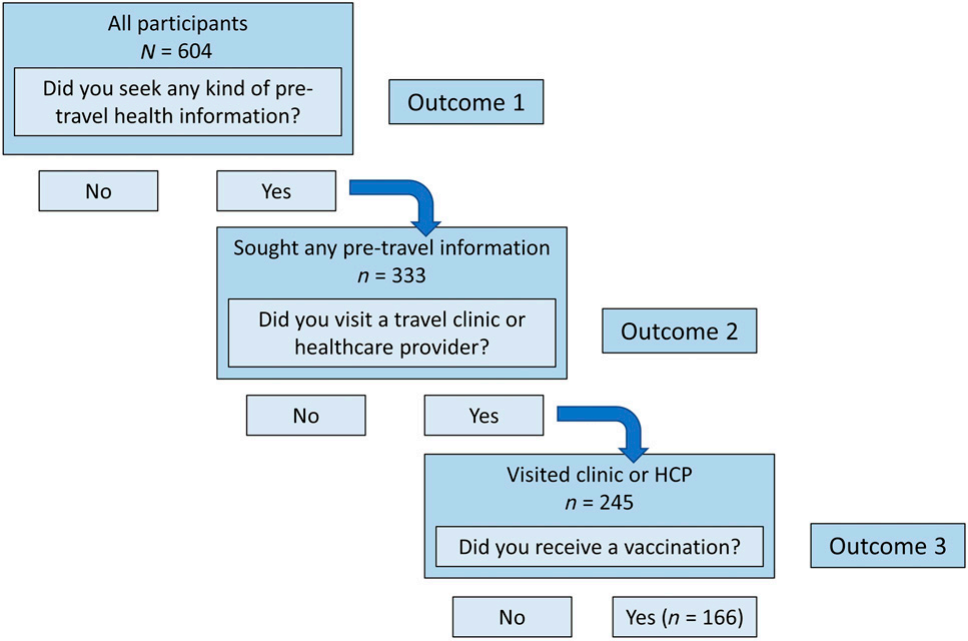
Pretravel health care–seeking decision pathway. Participants were analyzed in three nested groups, each associated with an outcome of interest. HCP = health care provider.

The survey population was subset into three nested groups based on the pretravel health-seeking decision pathway (Figure 2): full population (Group 1), those who sought any health information (Group 2), and those who visited a clinic or HCP (Group 3). Participants were included in Group 2 if they indicated that they gathered information about “understanding of health-related risks to the specific location” and/or “understanding the availability of health care and access to medication at the specific location” before their trip. Group 3 comprised members of Group 2 who indicated that they had visited a “travel health clinic” or “doctor or other health care provider” for information on travel and travel health before traveling. Each of these groups was analyzed separately for factors influencing three main outcomes of interest along the pre-travel health-seeking pathway: 1) sought health information, 2) visited a clinic, and 3) received a recommended or required vaccine (excluding influenza vaccine). Outcomes were coded as a binary response and modeled by logistic regression.

Survey questions and responses were reviewed and categorized according to the HBM. These categories were determined by consensus of the authors; a general breakdown of the type of question in each category can be found in Figure 1. When possible, variables with continuous or ordinal responses (often a scale of 1–7) were consolidated into two or three categories to reduce the dimensionality of the dataset.

### Factor analysis.

Because of the large number of variables in some HBM groups, we used factor analysis to derive index variables for use in logistic regression models. Factor analysis reduces groups of similar variables into factors that can then be defined based on which variables were included and used as an index for those variables. Maximum likelihood factor analysis with varimax rotation was done separately on the variables in each HBM category for all three study groups. Factors that explained ≥ 50% of cumulative variation were selected to be index variables, which were defined based on the individual loading score of each variable on the factor. For example, the first factor identified from the susceptibility category for outcome 1 had three variables with loading scores > 0.2: “born outside of United States,” “one or both parents born outside the United States,” and “immediate family living outside of United States.” These three variables were defined together as the index variable “family/self born outside of the U.S.” (Supplemental Table 3), which was then used in the regression analysis.

Factor analysis was performed on the susceptibility and self-efficacy categories for all three study groups and on benefits for Groups 2 and 3. Factor analysis was not appropriate (either too few variables or no factors identified) for the remaining HBM categories, so all relevant survey response variables for these categories were included in the regression analysis.

### Logistic regression.

The three outcome variables described above were coded as binary decision points in an individual’s pre-travel health-seeking pathway. To assess how different combinations of HBM categories influenced each outcome, we adopted the multi-model framework, under which models were defined a priori, assembled as a function of one or more HBM variable groups, demographic variables, and first-order interactions (Supplemental Table 2), and analyzed separately by logistic regression. The relative explanatory power of each model was then evaluated by comparing Akaike’s Information Criterion (AIC) score. The AIC score is a measure of the variability explained by a model, with a penalty that increases with the number of variables included in the model. Lower scores indicate a better model fit.

All models were analyzed separately by logistic regression. The AIC scores were tabulated and compared with a full model (including all variables considered) to determine the relative fit of each model for each outcome.

After fitting all a priori defined models, we conducted a stepwise fitting procedure based on the AIC score to determine the best possible model for each outcome given the available variables. This step AIC model summarizes the residual variability not explained by the predefined models.

All analyses were performed using R v.4.0.5 (R Foundation for Statistical Computing, Vienna, Austria). Significance of individual effects were evaluated at *P* < 0.05.

## RESULTS

Of 1,007 survey respondents, 604 had traveled to at least one at-risk country in the last 3 years (Supplemental Table 1). Study participants went on a total of 2,211 trips in the prior 3 years, the majority of which were to Asia (21%), Europe (19%), and Africa (12%). Participants were majority white (63%), male (57%), aged 30–54 years (49%), and had attended some postsecondary education below a master’s level (56%). These proportions remained approximately consistent across all three nested study groups.

Among all 604 participants, 333 (55%) reported that they sought any type of health information; health information seeking was defined by participants indicating they had sought information about “understanding of health-related risks to the specific location” and/or “understanding the availability of health care and access to medication at the specific location” before traveling. Of these 333, 245 (41% of total) reported visiting a travel clinic or HCP. Finally, of these 245, 166 (27% of total) reported receiving a vaccine before traveling (Table 1, Figure 2).

**Table 1:** Population demographics

Demographic	Outcome 1: Sought any health information			Outcome 2: Visited clinic or HCP			Outcome 3: Received vaccine		
	*n*	Yes	%	*n*	Yes	%	*n*	Yes	%
Total	604	333	55	333	245	74	245	166	68
Race									
Nonwhite	221	107*	48	107	74	69	74	51	69
White	383	226*	59	226	171	76	171	115	67
Gender									
Male	347	200	58	200	151	76	151	106	70
Female	257	133	52	133	94	71	94	60	64
Age, years									
Under 30	127	66	52	66	53	80	53	33	62
30–54	295	160	54	160	108	68	108	72	67
55+	182	107	59	107	84	79	84	61	73
Education									
High school or less	38	18	47	18	12	67	12	5*	42
At least some college	341	181	53	181	135	75	135	87*	64
Advanced degree	225	134	60	134	98	73	98	74*	76

HCP = health care provider. Chi-squared analysis was used to determine correlations between demographic and outcome variables.

* *P* < 0.05.

White participants were more likely to seek any type of health information than nonwhite participants (*P* = 0.015). Likelihood of vaccination also differed by education level, with higher levels of education significantly associated with a greater proportion of vaccinated participants (*P* = 0.029). The relationship between age and visiting a clinic or HCP also approached significance (*P* = 0.052); the middle-aged group (aged 30–54 years) was least likely to visit a clinic or HCP (68% versus about 80% for the younger and older groups). Differences between all other demographic groups were not statistically significant (Table 1).

### Modeling decision outcomes.

#### Outcome 1: Sought any kind of pretravel health information.

In general, models that contained the Susceptibility and Benefits HBM categories were the best predictors of health information seeking (outcome 1). No predefined HBM model was a better predictor of outcome 1 than the full model; however, the model “travelers’ peace of mind” (susceptibility and benefits) was approximately equivalent in predictive value to the full model (Table 2).

**Table 2:** Model descriptions and AIC values

Model name	AIC value			HBM categories included				
	Outcome 1: sought any health information	Outcome 2: visited clinic or HCP	Outcome 3: received vaccine	SUS	SEV	BEN	BAR	SEF
Full model	806.21	374.78	319.25	SUS	SEV	BEN	BAR	SEF
Demographics only	839.07	386.30	311.47	–	–	–	–	–
Perceived disease risk	809.83	377.28	315.28	SUS	SEV	BEN	–	–
Healthy lifestyle	–	384.26	313.80	–	–	–	–	SEF
Cost concerns	–	384.67	313.43	–	–	–	BAR	–
Travelers’ peace of mind	806.82*	370.79*	310.56	SUS	–	BEN	–	–
Proactive travel planner	829.46	372.85	313.61	SUS	–	–	–	SEF
Vaccine unnecessary/ineffective	822.08	380.22	307.88*	–	–	BEN	–	–
Severity of disease	831.66	391.82	314.11	–	SEV	–	–	–
Value of vaccine	–	–	310.91	–	–	BEN	BAR	–
Step AIC	787.07	363.69	307.36	–	–	–	–	–

AIC = Akaike’s Information Criterion; BAR = Perceived Barriers; BEN = Perceived Benefits; HBM = Health Belief Model; SEF = Self-Efficacy; SEV = Perceived Severity; SUS = Perceived Susceptibility. Models were predetermined and analyzed using logistic regression. The step AIC best fit model was determined using a stepwise fitting procedure. Smaller values for AIC value indicate a better fit. All variables included in each model can be seen in Supplemental Table 2.

* Best fit model for outcome, not including step AIC model.

In the full model, the only significant predictors (*P* < 0.05) of information seeking were “cautious on this trip” (Susceptibility), “non-vaccine precautions sufficient” (Benefits), and “budgeted for travel health” (Barriers). Similarly, in the travelers’ peace of mind model, cautious on this trip and non-vaccine precautions sufficient were the only significant predictors of information seeking. Cautious on this trip is an index variable capturing an individual’s level of concern about security, food and water safety, and potential for illness during their most recent trip to an at-risk country. Non-vaccine precautions sufficient was defined by participants’ level of agreement with the statement “I do not need travel vaccines if I take proper precautions and preventative treatments.” In both models, cautious on this trip was positively associated with health information seeking and non-vaccine precautions sufficient was negatively associated.

In the stepwise model, representing the best possible predictive value, an approximately equal number of variables from each of the HBM categories were retained. Again, cautious on this trip, and non-vaccine precautions sufficient were the most influential variables. No demographic variables were significant in either this model or in the demographics-only model (Table 3).

**Table 3:** Regression output for full, best fit, and step AIC models

Outcome 1: Sought any pre-travel care		Full model		Best fit model†		Step AIC	
Variable	HBM cat.	Estimate		Estimate		Estimate	
Intercept	–	−1.341		−0.462		−1.230	**
Family/self born outside U.S.	SUS	−0.005		−0.045		0.184	
Generally cautious traveler	SUS	0.203	*	0.217	*	–	
Cautious on this trip	SUS	0.350	***	0.422	****	0.352	***
History of health challenges	SEV	0.294		–		0.313	
Cost outweighs risk	SEV	−0.178		–		−0.173	
Disease is treatable	SEV	0.059		–		–	
Disease is not severe	SEV	−0.037		–		–	
Vaccine benefits outweigh risk	BEN	−0.004		0.017		–	
Non-vaccine precautions sufficient	BEN	−0.397	****	−0.409	****	−0.439	****
Vaccine does not work	BEN	−0.287		−0.247		–	
Food/water precautions sufficient	BEN	0.425	*	0.387	*	0.401	*
Confident will not get sick	BEN	0.390	*	0.389	*	0.444	**
Feels safer with vaccine	BEN	0.220		0.251		–	
Doesn’t want to miss out due to illness	BEN	0.340	*	0.372	*	0.386	**
Budgeted for travel health	BAR	0.493	**	–		0.491	**
Cost concern	BAR	0.274		–		–	
Proactive about health care	SEF	0.047		–		–	
Healthy diet and exercise	SEF	0.161		–		0.169	*
Male	DEMO	−0.295		−0.278		–	
Age 55+	DEMO	−0.082		−0.054		–	
Age < 30	DEMO	−0.281		−0.253		–	
Male, age 55+ interaction	DEMO	0.274		0.218		–	
Male, age < 30 interaction	DEMO	0.456		0.362		–	

Outcome 2: Visited clinic or HCP		Full model		Best fit model‡		Step AIC	
Variable	HBM cat.	Estimate		Estimate		Estimate	
Intercept	–	0.566		0.811	****	0.237	
Family/self born outside U.S.	SUS	−0.496	***	−0.515	****	−0.521	****
Generally cautious traveler	SUS	−0.008		−0.021		–	
Cautious on this trip	SUS	0.125		0.243		–	
History of health challenges	SEV	−0.308		–		–	
Cost outweighs risk	SEV	−0.079		–		–	
Disease is treatable	SEV	−0.166		–		–	
Disease is not severe	SEV	0.485		–		–	
Non-medical precautions sufficient	BEN	−0.579	***	−0.505	***	−0.558	***
Doesn’t want to disrupt trip	BEN	0.200		0.180		–	
Budgeted for travel health	BAR	0.478		–		0.412	
Cost concern	BAR	−0.185		–		–	
Healthy diet and exercise	SEF	0.324	*	–		0.357	**
Proactive about health care	SEF	0.263		–		0.242	
Male	DEMO	0.291		0.179		0.192	
Age 55+	DEMO	0.582		0.485		0.557	*
Age < 30	DEMO	1.425	***	1.327	***	1.398	***
Male, age 55+ interaction	DEMO	−0.639		−0.505		−0.489	
Male, age < 30 interaction	DEMO	−1.571	*	−1.372	*	−1.598	**

Outcome 3: Received vaccine		Full model		Best fit model§		Step AIC	
Variable	HBM cat.	Estimate		Estimate		Estimate	
Intercept	–	3.004	***	1.131	****	1.981	****
Family/self born outside U.S.	SUS	−0.245		–		−0.265	*
History of travel illness	SUS	0.219		–		–	
Generally cautious traveler	SUS	−0.064		–		–	
History of health challenges	SEV	−0.242		–		–	
Cost outweighs risk	SEV	−0.195		–		–	
Disease is treatable	SEV	−0.011		–		–	
Disease is not severe	SEV	−0.749		–		0.319	**
Doesn’t want to disrupt trip	BEN	0.266		0.331	**	0.817	*
Non-medical precautions not sufficient	BEN	0.290		0.333	*	0.340	*
Budgeted for travel health	BAR	0.073		–		–	
Cost concern	BAR	−0.357		–		–	
Healthy diet and exercise	SEF	0.237		–		0.236	
Proactive about health care	SEF	0.096		–		–	
Male	DEMO	−0.660		−0.769	*	−0.695	
Age 55+	DEMO	−0.329		−0.183		−0.309	
Age < 30	DEMO	−0.607		−0.762	*	−0.673	
Male, age 55+ interaction	DEMO	0.610		0.573		0.616	
Male, age < 30 interaction	DEMO	1.874	**	1.995	**	1.890	**

AIC = Akaike’s Information Criterion; BAR = Perceived Barriers; BEN = Perceived Benefits; cat = category; HBM = Health Belief Model; SEF = Self-Efficacy; SEV = Perceived Severity; SUS = Perceived Susceptibility. Models were predetermined and analyzed using logistic regression with a binary outcome. Outcome 1 was analyzed for Group 1, outcome 2 for Group 2, and outcome 3 for Group 3 (Figure 2). The step AIC best fit model was determined using a stepwise fitting procedure. All variables included in each model can be seen in Supplemental Table 2. The best fit model for each outcome is listed below.

* *P* < 0.1; ** *P* < 0.05; *** *P* < 0.01; **** *P* < 0.001.

† Best fit model outcome 1: Travelers’ peace of mind.

‡ Best fit model outcome 2: Travelers’ peace of mind.

§ Best fit model outcome 3: Vaccine not necessary/ineffective.

#### Outcome 2: Visited a travel clinic or HCP.

Models that contained the susceptibility HBM category also tended to be the best predictors of visiting a clinic or HCP (outcome 2). Both the travelers’ peace of mind (susceptibility and benefits) and “proactive travel planner” (susceptibility and self-efficacy) models were better fits to the data than the full model (Table 2); of the two, travelers’ peace of mind was the better fit.

In both the full model and travelers’ peace of mind, family/self born outside the U.S. (susceptibility), “non-medical precautions sufficient” (Benefits), and “age < 30” were the most significant predictors of visiting a clinic or HCP. Family/self born outside the U.S. is an index variable capturing whether the respondent or respondent’s family had been born or were living abroad. Non-medical precautions sufficient is an index variable influenced by positive responses to non-vaccine precautions sufficient and “food and water precautions sufficient,” and negative responses to “feels safer with vaccine.” Both family/self born outside the U.S. and non-medical precautions sufficient were negatively associated with visiting a clinic, whereas age < 30 was positively associated.

The stepwise model contained variables from all the HBM categories except severity, with the same three most significant variables as the full model and travelers’ peace of mind (Table 3).

#### Outcome 3: Received vaccine.

Models predicting whether an individual received a vaccine prior to traveling (outcome 3) fit the data best when they contained the benefits HBM category. The model containing only benefits and demographics—“vaccine not necessary”—was only marginally worse than the stepwise model (ΔAIC < 1) (Table 2). This model contained only the two index variables defined for the Benefits HBM category and the demographic variables. Only “doesn’t want to disrupt trip” and the interaction between male and age < 30 were significant. Doesn’t want to disrupt trip is an index variable that includes the variables “doesn’t want to miss out due to illness” and “doesn’t want illness to interrupt trip.” For both these variables, participants were asked “Which of the following are reasons why you would consider receiving the [cholera] vaccine?” Both doesn’t want to disrupt trip and male age < 30 were positively associated with the outcome.

The stepwise model for outcome 3 contained variables from all HBM categories except barriers. Only “disease is not severe” (defined by participants responding “not concerned about severity of disease” when asked why they chose not to get vaccinated) and the male age < 30 interaction were significant. Again, both variables were positively associated with receiving a vaccine. The only other non-demographic variable retained in this model was “non-medical precautions not sufficient,” an index variable influenced by negative responses to non-vaccine precautions sufficient and food and water precautions sufficient and positive responses to feels safer with vaccine. This variable was positively associated but was not significant (*P* = 0.084) (Table 3).

## DISCUSSION

In our study of 604 individuals who traveled in the past 3 years to a country at risk for a VPD, 55% reported seeking pretravel health information, a value consistent with previous findings.^3^ Limited information in the literature makes it difficult to corroborate our finding that 41% of the total study population (74% of those who sought any information); one study of US travelers to LMICs found that 38% of travelers who sought any pre-travel health information visited their primary care provider and 30% visited a travel medicine specialist, however there may have been overlap between these two groups.^3^ Among those who visited a clinic or HCP, 68% reported actually receiving a vaccine. Although not exactly comparable, this is similar to previous findings that approximately 25% of travelers who see a provider before traveling refuse at least one vaccine.^4^ Although these results suggest that our population is fairly representative of U.S.-based international travelers, future work should take a more deliberate approach to selecting a survey population.

Of the seven predetermined models examined in this study, travelers’ peace of mind, containing the HBM categories susceptibility and benefits, was effective in predicting both outcome 1 (pre-travel health information seeking) and outcome 2 (visiting a clinic or HCP). Proactive travel planner (susceptibility and self-efficacy) was also effective in predicting outcome 2. The model “vaccine is not effective,” containing only the Benefits HBM category, was most effective in predicting outcome 3 (receiving a vaccine) (see Figure 1 for the decision-making pathway).

In general, models containing variables from the susceptibility and benefits categories were most successful in predicting all three outcomes, which suggests that these may be more relevant factors in pre-travel health decision-making than the other HBM categories. A 2021 study of COVID-19 vaccination also found susceptibility and benefits to be most relevant, but almost every combination of HBM categories can be found in the literature.^9^^–^^14^ Given this lack of consensus, it is difficult to say if these results are consistent with other findings. Susceptibility was a more relevant consideration earlier in the decision-making pathway (outcomes 1 and 2), but was less relevant later on (outcome 3). The Susceptibility category consists of questions about people’s perception of their own health, their experience with travel and/or life abroad, and their attitudes toward travel health and preparedness. If perceived susceptibility is the most salient factor in determining pre-travel health care seeking, this may present an opportunity to target messaging to travelers that emphasizes the individual’s actual likelihood of contracting a travel-associated infectious disease.

The Benefits category was highly relevant for all three outcomes. Benefits consisted of questions about the utility of vaccination and other pre-travel health care. The relevance of this category suggests that emphasizing the effectiveness of vaccination and other travel health care may inspire more pre-travel health-seeking behavior and vaccine uptake. A large proportion of our study population (45%) did not seek any information before traveling. These may have been individuals who were confident enough in their travel experience to not consider it necessary, including people who travel regularly to the same region for work or other reasons and those who are traveling to their home country to visit friends or relatives (VFR travelers). A VFR traveler in particular is known to seek travel health care less often than non-VFR travelers, which may contribute to this pattern; 24% of respondents to this survey reported having one or more parents born outside of the United States, though it is not clear from the survey questions whether they were traveling back to their region of birth or elsewhere.^16^

It is interesting to note the discrepancy between the number of travelers who sought health information and those who actually went to a clinic or HCP; only half of respondents reported seeking any health information, but 74% of those who sought information went on to visit a clinic. This suggests that information available on the Internet and from other non-HCP sources does a good job of encouraging travelers to see a doctor before they travel (though there is likely a correlation between the type of person who seeks information online and the type who sees a doctor before traveling). This finding fits with our assessment that HBM categories influenced by providing information about a given disease, such as susceptibility and benefits, are more relevant in decision-making than the more individual-based categories, such as barriers (e.g., cost) and self-efficacy; if seeking health information provides a more accurate estimate of susceptibility and benefits, it makes sense that a disproportionately high percentage of individuals who seek information would go on to seek care. A large study of U.S. travelers had similar findings; when asked why they were refusing a travel vaccine, more than 60% of respondents cited low perceived susceptibility.^4^

Meanwhile, the 26% of participants who sought information but did not visit a clinic may have felt the information they received did not necessitate a doctor visit, may have already received suggested vaccines and/or considered themselves immune owing to past exposures, or may have faced a barrier to care (cost, location, knowledge of resources, etc.).^3^ Understanding this group is important to identifying key areas for outreach, and future studies should ask questions targeting this decision.

Yellow fever vaccine requirements play an important role in determining an individual’s likelihood of visiting a clinic and getting vaccinated. For individuals traveling to yellow fever endemic or partially endemic regions, vaccination rates among those who go to a clinic or HCP is upward of 90%. Of the 30 countries included in our study, six required a yellow fever vaccination; however, the survey did not identify which country each individual had traveled to, so we were unable to adjust for this requirement.^17^

The survey used here was designed for market research rather than public health analysis. Although certain questions were relevant to our study, most were either irrelevant or required too many unvalidated assumptions to include in our analysis. The survey’s design also limited the size of our final analysis sample. The original population reached by this survey was more than 1,000, but because of the skip logic embedded in the survey and the discordant goal of the survey design, many of these participants were routed to a set of questions that was not relevant to this study and therefore could not be included in our analysis. In addition, the survey we used here had more than 150 questions, several of which contained 30 or more sub-questions; the decision of which questions to include and how to categorize each into an HBM group was therefore subjective. Future surveys should use the results of our study as a framework to examine these relationships more deeply and provide context for improving clinical practice by including specific questions about travel experience and medical/vaccination history, as well as questions written specifically for each HBM category.

Despite these limitations, the results of this study paint a meaningful picture of our survey population’s motivations as they move along the pretravel health-seeking decision pathway. The results emphasize the importance of individual beliefs and perceptions, particularly in relation to the individual’s perception of his or her susceptibility to disease and the benefit of interventions, in predicting pretravel health behaviors. Understanding this interaction has the potential to help shape the way HCPs and public health entities reach out to travelers and encourage health care seeking and vaccine uptake.

## Supplemental Materials


Supplemental materials
