# Supplementary material for: Using the Health Belief Model to Predict Pre-Travel Health Decisions among U.S.-Based Travelers
Source: Am J Trop Med Hyg. 2023 Sep 5;109(4):937–44. doi: 10.4269/ajtmh.22-0633 (PMC10551073; doi:10.4269/ajtmh.22-0633)
Supplement: Supplementary file 1 [file tpmd220633.SD1.pdf]

Table S1. List of destination countries included in this analysis

COUNTRY	REGION
Angola	Africa
Bangladesh	Asia
Bhutan	Asia
Burundi	Africa
Cambodia	Asia
Cameroon	Africa
Chad	Africa
Democratic Republic of the Congo	Africa
Ethiopia	Africa
Ghana	Africa
Haiti	Caribbean/other
India	Asia
Indonesia	Asia
Kenya	Africa
Laos	Asia
Malawi	Africa
Mozambique	Africa
Namibia	Africa
Nepal	Asia
Nigeria	Africa
Philippines	Asia
Rwanda	Africa
Senegal	Africa
Somalia	Africa
South Sudan	Africa
Tanzania	Africa
Uganda	Africa
Yemen	Middle east
Zambia	Africa
Zimbabwe	Africa

Table S2. All variables included in each regression model

Models were determined a priori based on hypothesized reasons that a traveler may make a certain pre-travel health decision. Variables were sorted into HBM Categories at the discretion of the authors. Variables are color coded based on HBM category; SUS: Perceived Susceptibility; SEV: Perceived Severity; BEN: Perceived Benefits; BAR: Perceived Barriers; SEF: Self-Efficacy

Model	***Sought any health info***	***Visited clinic or HCP***	***Received vaccine***
Full<br>- SUS<br>- SEV<br>- BEN<br>- BAR<br>- SEF	• family/self born outside of US<br>• generally cautious traveler<br>• cautious on this trip<br>• history of health challenges<br>• cost outweighs risk<br>• disease is treatable<br>• disease is not severe<br>• vaccine benefits outweigh risk<br>• non-medical precautions sufficient<br>• vaccine does not work<br>• food and water precautions are sufficient<br>• confident will not get sick<br>• feels safer with vaccine<br>• doesn't want to miss out due to illness<br>• budgeted for travel health<br>• cost concern<br>• healthy diet and exercise<br>• proactive about health care<br>• age<br>• gender<br>• age*gender	• family/self born outside of US<br>• generally cautious traveler<br>• cautious on this trip<br>• history of health challenges<br>• cost outweighs risk<br>• disease is treatable<br>• disease is not severe<br>• doesn't want to disrupt trip<br>• non-vaccine precautions sufficient<br>• budgeted for travel health<br>• cost concern<br>• healthy diet and exercise<br>• proactive about health care<br>• age<br>• gender<br>• age*gender	• family/self born outside of US<br>• generally cautious traveler<br>• cautious on this trip<br>• history of health challenges<br>• cost outweighs risk<br>• disease is treatable<br>• disease is not severe<br>• doesn't want to disrupt trip<br>• non-vaccine precautions sufficient<br>• budgeted for travel health<br>• cost concern<br>• healthy diet and exercise<br>• proactive about health care<br>• age<br>• gender<br>• age*gender
Demographics	• age<br>• gender<br>• age*gender	• age<br>• gender<br>• age*gender	• age<br>• gender<br>• age*gender
Perceived disease risk	• family/self born outside of US<br>• generally cautious traveler<br>• cautious on this trip<br>• history of health challenges<br>• cost outweighs risk<br>• disease is treatable<br>• disease is not severe	• family/self born outside of US<br>• generally cautious traveler<br>• cautious on this trip<br>• history of health challenges<br>• cost outweighs risk<br>• disease is treatable<br>• disease is not severe	• family/self born outside of US<br>• generally cautious traveler<br>• cautious on this trip<br>• history of health challenges<br>• cost outweighs risk<br>• disease is treatable<br>• disease is not severe

	• vaccine benefits outweigh risk<br>• non-medical precautions sufficient<br>• vaccine does not work<br>• food and water precautions are sufficient<br>• confident will not get sick<br>• feels safer with vaccine<br>• doesn't want to miss out due to illness<br>• age<br>• gender<br>• age*gender	• doesn't want to disrupt trip<br>• non-vaccine precautions sufficient<br>• age<br>• gender<br>• age*gender	• doesn't want to disrupt trip<br>• non-vaccine precautions sufficient<br>• age<br>• gender<br>• age*gender
Healthy lifestyle	--	• healthy diet and exercise<br>• proactive about health care	• healthy diet and exercise<br>• proactive about health care
Cost concerns	--	• budgeted for travel health<br>• cost concern	• budgeted for travel health<br>• cost concern
Travelers' peace of mind	• family/self born outside of US<br>• generally cautious traveler<br>• cautious on this trip<br>• vaccine benefits outweigh risk<br>• non-medical precautions sufficient<br>• vaccine does not work<br>• food and water precautions are sufficient<br>• confident will not get sick<br>• feels safer with vaccine<br>• doesn't want to miss out due to illness<br>• age<br>• gender<br>• age*gender	• family/self born outside of US<br>• generally cautious traveler<br>• cautious on this trip<br>• doesn't want to disrupt trip<br>• non-vaccine precautions sufficient<br>• age<br>• gender<br>age*gender	• family/self born outside of US<br>• generally cautious traveler<br>• cautious on this trip<br>• doesn't want to disrupt trip<br>• non-vaccine precautions sufficient<br>• age<br>• gender<br>• age*gender
Proactive travel planner	• family/self born outside of US<br>• generally cautious traveler<br>• cautious on this trip<br>• healthy diet and exercise<br>• proactive about health care	• family/self born outside of US<br>• generally cautious traveler<br>• cautious on this trip<br>• healthy diet and exercise<br>• proactive about health care	• family/self born outside of US<br>• generally cautious traveler<br>• cautious on this trip<br>• healthy diet and exercise<br>• proactive about health care
Vaccine not necessary/ineffective	• vaccine benefits outweigh risk<br>• non-medical precautions sufficient<br>• vaccine does not work<br>• food and water precautions are	• doesn't want to disrupt trip<br>• non-vaccine precautions sufficient<br>• age<br>• gender	• doesn't want to disrupt trip<br>• non-vaccine precautions sufficient<br>• age<br>• gender

	sufficient<br>• confident will not get sick<br>• feels safer with vaccine<br>• doesn't want to miss out due to illness<br>• age<br>• gender<br>• age*gender	• age*gender	• age*gender
Severity of disease	• history of health challenges<br>• cost outweighs risk<br>• disease is treatable<br>• disease is not severe<br>• age<br>• gender<br>• age*gender	• history of health challenges<br>• cost outweighs risk<br>• disease is treatable<br>• disease is not severe<br>• age<br>• gender<br>• age*gender	• history of health challenges<br>• cost outweighs risk<br>• disease is treatable<br>• disease is not severe<br>• age<br>• gender<br>• age*gender
Value of vaccine	--	--	• doesn't want to disrupt trip<br>• non-vaccine precautions sufficient<br>• budgeted for travel health<br>• cost concern<br>• age<br>• gender<br>• age*gender
Step AIC	• generally cautious traveler<br>• cautious on this trip<br>• history of health challenges<br>• cost outweighs risk<br>• non-medical precautions sufficient<br>• food and water precautions are sufficient<br>• confident will not get sick<br>• doesn't want to miss out due to illness<br>• budgeted for travel health<br>• proactive about health care<br>• healthy diet and exercise	• family/self born outside US<br>• non-vaccine precautions sufficient<br>• budgeted for travel health<br>• healthy diet and exercise<br>• proactive about health care<br>• age<br>• gender<br>• age*gender	• family/self born outside US<br>• disease is not severe<br>• doesn't want to disrupt trip<br>• non-vaccine precautions sufficient<br>• healthy diet and exercise<br>• age<br>• gender<br>• age*gender

Table S3. Loading scores for factor analysis

Maximum likelihood factor analysis with varimax rotation was done separately on the variables in each HBM category for all three study groups. Variables of interest are shown on the left side of the table, while the index variables indicated by factor analysis are shown at the top. For clarity only loading scores > 0.2 are shown. Factors that explained ≥ 50% of cumulative variation were selected to be index variables; only indices chosen as index variables are displayed. The Severity and Barriers HBM categories did not contain enough variables to be appropriate for factor analysis.

	Index Variables								
	**Sought any health info**			**Visited clinic or HCP**			**Received vaccine**		
Susceptibility variables	family/self born outside of US	generally cautious traveler	cautious on this trip	family/self born outside of US	generally cautious traveler	cautious on this trip	family/self born outside of US	history of travel illness	generally cautious traveler
overall good health									
history of health challenges								0.22	
worries about health		0.41			0.39			0.29	0.39
cautious in choosing countries to travel to		0.64			0.62				0.57
willing to cancel trip in face of emerging risk		0.61			0.55				0.55
history of bad travel experiences		0.63			0.61				0.61
typical number of trips per year									
budgeted for travel health			0.22						
concerned about security at the destination		0.21	0.50			0.49			
concerned about safety of food, water, and sanitation at the destination			0.67			0.67			
concerned about potential for sickness while traveling			0.67			0.60			
familiarity with travel illness (cholera)									

has contracted any travel illness while abroad								0.60	
knows anyone who has contracted travel illnesses while traveling abroad								0.74	
other precautions (non vaccine) are sufficient		0.25			0.24				0.21
risk of contracting illness is low									
disease is treatable									
disease is not severe									
food and water precautions are sufficient									
feels safer with vaccine									
risk of disease is high									
food and water may not be safe								0.33	
born outside of United States	0.69			0.60			0.54		
one or both parents born outside of United States	0.82			0.84			0.83		
immediate family living outside of United States	0.63			0.67			0.58		
Benefit variables				doesn't want to disrupt trip	non-medical precautions sufficient		doesn't want to disrupt trip	non-medical precautions not sufficient	
benefits of vaccine outweigh risk					-0.22			0.27	
non-vaccine precautions sufficient				-0.26	0.53		-0.3	-0.42	
vaccine is not effective					0.23			-0.25	
food and water precautions are sufficient					0.39			-0.41	

doesn't want illness to interrupt trip				0.31			0.34	0.35	
feels safer with vaccine				0.23	-0.42		0.29	0.5	
doesn't want to miss out due to illness				1.00			1.00		
Self-efficacy variables	healthy diet and exercise	proactive about healthcare		healthy diet and exercise	proactive about healthcare		healthy diet and exercise	proactive about healthcare	
does not hesitate to visit a doctor when sick		0.29			0.21			0.25	
stays up to date on health advice	0.28				0.58			0.61	
asks questions about doctor recommendations and prescriptions					0.46			0.47	
trusts own judgement in health decisions					0.25			0.23	
proactive in preventing health problems	0.36				0.54		0.22	0.66	
prioritizes healthy diet	0.85			0.71	0.44		0.98		
prioritizes organic food	0.47			0.38	0.37		0.45		
prioritizes full nights rest	0.38			0.55			0.25		
prioritizes routine doctor visits		0.99		0.23					
prioritizes regular exercise	0.50			0.48	0.35		0.37	0.32	
prioritizes relaxation	0.23			0.47					
prioritizes maintaining up to date vaccinations		0.47		0.22					
proactively educates self on travel risks									
